# Difficult phylogenetic questions: more data, maybe; better methods, certainly

**DOI:** 10.1186/1741-7007-9-91

**Published:** 2011-12-29

**Authors:** Hervé Philippe, Béatrice Roure

**Affiliations:** 1 Département de Biochimie, Centre Robert-Cedergren, Université de Montréal, Succursale Centre-Ville, Montréal, Québec H3C 3J7, Canada

## Abstract

Contradicting the prejudice that endosymbiosis is a rare phenomenon, Husník and co-workers show in *BMC Biology *that bacterial endosymbiosis has occured several times independently during insect evolution. Rigorous phylogenetic analyses, in particular using complex models of sequence evolution and an original site removal procedure, allow this conclusion to be established after eschewing inference artefacts that usually plague the positioning of highly divergent endosymbiont genomic sequences.

See research article http://www.biomedcentral.com/1741-7007/9/87

## Commentary

Modern Western civilisation has promoted individualism, individual autonomy and self-determination to such a high level that it has even permeated biological thought. For instance, based on the idea that individuals can and should thrive on their own, it is commonly accepted that endosymbiosis - an organism living non-autonomously inside another organism - is extremely difficult and has arisen very rarely throughout evolution. However, it is a Western prejudice that any organism, including humans, can thrive autonomously. This prejudice is contradicted by the data. For instance, a mere few percent of microorganisms are able to grow in pure culture. Obviously, an organism spends all its lifetime in close contact and interaction with many other organisms, so that a pure culture is likely to be an extremely hostile environment. In the era of metagenomics it is now common knowledge that in a human body, bacterial cells not only outnumber human cells but also provide numerous essential services. Accordingly, organisms have been selected to live in commensal or symbiotic relationships with one or several other species (this does not mean universal harmony; such interactions often evolve into parasitism). Endosymbiosis is simply a further twist of a very common phenomenon. Given that the prejudice inspired by individualism is erroneous, the recent demonstration that endosymbioses are more frequent than previously thought ought not to be but apparently is surprising [1]. This surprise reflects, however, our anthropocentrism rather than a basic conflict of our conception of biological relationships. A study published in *BMC Biology *[2] adds new evidence to support this by demonstrating that at least four independent endosymbioses of an enterobacterium within an insect have occurred.

## Systematic errors in endosymbiont phylogenies

Husník and co-workers [2] address the question of the evolution of endosymbiosis in insects by applying a phylogenomic approach - the use of complete genomes to infer phylogenetic relationships. A naïve opinion is that phylogenomics will end incongruence in phylogeny, and therefore that gathering more data will suffice to resolve outstanding phylogenetic questions. However, while the use of many genes does reduce stochastic errors (due to improved sample size), it simultaneously makes systematic errors more apparent [3]. Systematic errors are due to the limitations of tree inference methods, which do not sufficiently account for the complexity of the genomic data. As such, systematic errors will lead to more and more biased results as the amount of data increases, thus producing highly supported, yet erroneous, phylogenomic trees.

Preventing systematic errors is therefore the most important issue in phylogenomics. The principal cause of reconstruction artefacts is the difficulty of detection of multiple nucleotide substitutions occurring at a given site by inference methods. Three complementary approaches have been developed to reduce the impact of systematic errors (reviewed in [3]):

(1) the use of a large number of species, naturally easing the detection of multiple substitutions,

(2) the use of complex models of sequence evolution (especially by accounting for heterogeneity across sites and over time), allowing more accurate detection of multiple substitutions,

(3) the removal of the fastest evolving sites, which are obviously the most prone to exhibit multiple substitutions.

Inferring the origin of endosymbionts is typically difficult for phylogenomics. Their intracellular lifestyle introduces similar biases in independent endosymbiotic organisms, with such convergences leading to potentially erroneous grouping of unrelated species. More precisely, because of their small effective population size, endosymbionts are subject to an irreversible accumulation of deleterious mutations, known as Muller's ratchet, thereby evolving at an accelerated rate. These accelerations may lead to the well-known long branch attraction artefact, in which the longest branches of a phylogenetic tree are clustered together irrespective of their true relationships. Moreover, due to this inefficient purifying selection, endosymbionts are more sensitive to mutational bias, with their genomes becoming more A+T rich. The erroneous grouping of species with similar nucleotide composition is also a frequent artefact (e.g. [4]).

Husník *et al. *[2] took many precautions to reduce the effect of these two biases that favour, potentially erroneously, the clustering of endosymbionts. First, they selected all the genes that are single copy in the 50 complete genome sequences of γ-Proteobacteria, hence avoiding identification problems caused by multi-copy gene families. Second, they used as many enterobacterial species as are currently available, although they could have used more outgroup species. These trivial, sometimes neglected, steps lead to a large dataset of 69 genes (63,462 nucleotidic sites, or 21,154 amino acid sites). Not surprisingly, a naïve phylogeny based on nucleotides and assuming compositional homogeneity over time leads to the grouping of the fast evolving, A+T-rich, endosymbionts (named hereafter the FEAT group), with high statistical support. Although this topology is certainly partly incorrect (for example, the inclusion of two species with the highest AT content, *Riesia *and *Wigglesworthia*, within the genus *Buchnera*), the monophyly of most endosymbionts might be correct, since it is possible for a bias to reinforce a true (but unknown) phylogenetic signal.

## Handling the complexity of evolutionary processes is of prime importance

Given the impossibility of experimental validation in what is fundamentally an historical science, corroboration is the most efficient support of an inference [5]. In general, phylogenomicists look for congruence among independent sets of characters (for example, between primary sequences and gene content, gene order or intron positions). Alternatively, as done by Husník *et al. *[2], congruence on the same dataset among independent methods is also relevant, especially in the case of bacterial endosymbionts, for which other character types are non-existent or inadequate; for instance gene content is highly prone to convergence. Husník and colleagues [2] hence applied a variety of methods known to reduce artefacts due to compositional bias and/or long branch attraction. Importantly, the more accurate the method is, the fewer endosymbionts are grouped, which strongly argues for several independent endosymbioses.

The use of amino acid sequences is an effective way to reduce the misleading effect of nucleotide compositional heterogeneity, although some information is lost. The use of a standard site-homogeneous model leads to the exclusion of *Regiella *from the FEAT group, while the CAT+GTR model [6] that simultaneously handles heterogeneity in the evolutionary process across sites and among amino acid substitutions leads to the further exclusion of *Ishikawaella*. Since the CAT+GTR model fits the data better and is less sensitive to long branch attraction [7], this first result is in agreement with an artefactual nature of the FEAT group. As nucleotide heterogeneity may affect amino acid composition, Husník *et al. *[2] applied the Dayhoff recoding. This is a recoding of amino acids into the six main Dayhoff categories, such as grouping the positive amino acids arginine, histidine and lysine, and is known to reduce possible biases [8], again at the cost of information. Interestingly, in the resulting phylogeny, the insect endosymbionts explode into four monophyletic groups dispersed over the enterobacterial tree. The disaggregation of the FEAT group is similarly observed for the analysis of nucleotidic sequences after removal of third codon positions or RY-coding (purine/pyrimidine), and the use of an improved model of sequence evolution. In particular, the use of a non-homogeneous model [9], that is, a model that does not assume homogeneity of nucleotide composition over time, recovers a topology that is highly similar to the Dayhoff-recoded topology.

**Figure 1:**
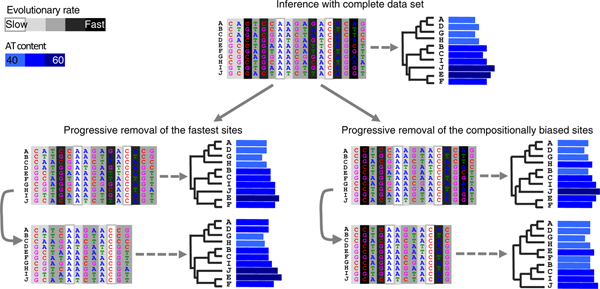
**Two different strategies of site removal to reduce systematic error**. Because the G+C content is heterogeneous across species, taxa E and F are erroneously recovered as a sister-group of taxon J in the phylogeny based on a phylogenomic dataset due to convergently acquired high G+C content. The standard approach consisting of removing the fastest evolving sites does not alleviate this artefact. The second strategy proposed by Husník *et al. *[2] consists of removing the positions that contain both A/T and G/C nucleotides, and thus are more likely to be compositionally biased. The method is more effective in recovering the correct topology (right side of the figure) when compositional bias is the main cause of systematic error.

To validate this result further, Husník and colleagues [2] applied another approach, the removal of sites. Unexpectedly, the classical removal of the fast evolving sites has little effect, leading simply to the exclusion of *Riesa *from the FEAT group. Indeed, under models accounting for the rate heterogeneity across sites, the likelihoods of a site requiring 20 substitutions on the incorrect topology and 22 substitutions on the correct topology are very similar for both topologies. Therefore the removal of the fastest evolving sites is expected to have limited benefit, even if these sites have more rapidly accumulated a deleterious compositional heterogeneity. Husník *et al. *[2] reasoned that, because of the high level of compositional heterogeneity, the most problematic sites might not be the fastest evolving sites, but the most compositionally biased. Accordingly, they decided to focus on sites that contain only adenine and thymine or guanine and cytosine, in other words, sites with a homogeneous A+T content. When they increasingly remove sites containing a large amount of both A/T and G/C, until only homogeneous sites remain (Figure 1), the FEAT group progressively disappears, in a very similar way to the result obtained with the model improvement discussed above. The reason is that a slowly evolving site with a non-homogeneous nucleotide composition can seriously bias phylogenetic inference: the likelihood of a site requiring 1 substitution on the incorrect topology and two substitutions on the correct topology is sensibly lower in the first case.

We recently obtained a similar result in the case of an animal phylogeny based on the mitochondrial genome. The removal of fast evolving sites has no effect, whereas the removal of heteropecillous sites, ones that change their substitution pattern over time, leads to the correct topology [10]. These two failures of fast site removal can be easily explained. Models of sequence evolution handle rate heterogeneity across sites anyway, usually through a gamma distribution, so that fast evolving sites will be detected, and have a limited effect on topology inference. In contrast, a site that violates model assumptions such as non-homogeneity of nucleotide composition across species might still evolve slowly and seriously impact phylogenetic reconstruction. The study of Husník *et al. *[2] and our work [10] argue in favour of developing methods that specifically remove model-violating sites rather than fast evolving sites.

Corroboration is key to solving difficult phylogenetic questions. Instead of using independent markers (for instance, from mitochondrion, plastid and nucleus), Husník *et al. *[2] successfully used three independent approaches to demonstrate that at least four endosymbioses of Enterobacteria have occurred in the insect lineage. More generally, this study demonstrates that, in spite of overwhelming genomic data, more effort should be put into refining data analysis. Unfortunately, the two approaches that are the most beneficial to phylogenetic accuracy - more species and better models - both imply a drastic increase in computation time. In a time of global warming and biodiversity loss, it is also urgent that scientists strive to decrease the environmental footprint of their research activities. Individualism is one cause of current environmental problems. An increase in our knowledge about the commonness of the symbiosis and its evolutionary advantages (by low consuming experiments) could be a way to change our societal paradigms and solve environmental crisis. The evolutionary advantages of endosymbioses should not be ignored.
